# Residential movement patterns of families of young children with chronic conditions in Ontario, Canada: a population-based cohort study

**DOI:** 10.1186/1475-9276-12-62

**Published:** 2013-08-20

**Authors:** Eyal Cohen, Nicole Yantzi, Jun Guan, Kelvin Lam, Astrid Guttmann

**Affiliations:** 1 Department of Pediatrics and Institute of Health Policy, Management & Evaluation, University of Toronto, Toronto, ON, Canada; 2 CanChild Centre for Childhood Disability Research, Hamilton, ON, Canada; 3 Department of Environmental Studies, Laurentian University, Sudbury, ON, Canada; 4 Institute for Clinical Evaluative Sciences, Toronto, ON, Canada; 5 Division of Pediatric Medicine, The Hospital for Sick Children, Department of Pediatrics, University of Toronto, 555 University Avenue, Toronto, ON M5G 1X8, Canada

**Keywords:** Movement, Chronic diseases, Complex chronic conditions, Socio-economic, Health, Health services research, Socioeconomic status, Chronic conditions, Geography, Children

## Abstract

**Introduction:**

Care giving for children with chronic diseases can lead to financial strain and compromised family well being. Little is known about whether these stresses lead to changes in residential movement patterns as they relate to income adequacy and proximity to care.

**Methods:**

We compared the residential movement patterns and associated changes in neighbourhood income of children with mild to severe chronic diseases compared with those that are healthy. A cohort of infants born from 2002–2007 in Ontario, Canada was followed for 5 years and divided into those with single- or multiple- body system complex chronic conditions (CCCs); low birth weight (LBW); asthma/recurrent wheeze (A/RW) and the control group of otherwise healthy children.

**Results:**

Of 598,716 children studied, 15,207 had a single CCC, 3,600 multiple CCCs, 33,206 LBW, 57,137 A/RW and 489,566 were healthy. Lowest income quintile children were most likely to move residence. Compared with healthy controls, chronic disease cohorts, apart from those with asthma, were more likely to be born in the lowest income quintile neighbourhood and to move. Among children who moved, all chronic disease cohorts were significantly more likely to move to a low income quintile neighborhood (adjusted odds ratios for all chronic disease cohorts of 1.1-1.2). There were no differences across cohorts in residential movement close to a children’s hospital.

**Conclusions:**

Young children with chronic conditions, particularly those born in low income neighbourhoods, are more likely to move residence than other healthy young children. However, it does not seem that proximity to specialized care is driving this movement. Further research is required to determine if these movement patterns impact the ability of children with chronic conditions to secure health services.

## Introduction

Over the past four decades, the number of children with a chronic illness has quadrupled [1]. Accordingly, improving health outcomes for these children and their family caregivers has become an important priority in contemporary health policy [2]. Previous studies have shown that families of children with chronic conditions experience major effects from the burden of care [3,4], including increased financial pressures [5,6]. Although cross-sectional studies have shown that families of children with chronic diseases are more likely to be of lower socio-economic status (SES) [7], it has never been demonstrated on a population level to what extent this occurs after a child is born with a chronic condition, as opposed to being a risk factor for having a newborn child with a chronic disease.

Equitable access to health services, especially tertiary and specialized care, is another key outcome for families of children with chronic conditions. Apart from traditional barriers such as health insurance, even in countries with universal insurance, other barriers exist. Researchers and policy makers have traditionally examined this for different populations in terms of geographic location of the service and individuals [8]. Key service barriers include travel time, distance and transportation [9,10]. To date there is a paucity of literature examining whether individuals/families of children with chronic diseases (lasting at least 12 months) and/or complex diseases (involving several different organ systems or 1 organ system requiring a high level of specialty care and hospitalization) move to new neighbourhoods to have better access to key health care services [11]. This issue is particularly relevant to families with children with chronic and/or complex conditions as distance from specialized, comprehensive care has been shown to affect family functioning [12]. However, it is not known whether families move to areas close to specialized care to mitigate these potential problems.

In deciding whether to move or not, individuals and families evaluate a diverse and complex array of push and pull factors such as employment prospects, educational opportunities, access to social support and recreational opportunities [13]. Depending on the context, a factor can push individuals/families from one residence into another or pull individuals/families to remain in their current residence. Health can be a push factor in terms of moving closer to caregivers [11,14]. At the same time health can also be a pull factor with numerous studies showing that individuals who migrate internationally report a higher health status compared to their remaining counterparts [15,16]. Families with children with chronic conditions may be faced with a number of push and pull factors including accessing health care and dealing with changing economic resources that impact their decision of whether or not to move.

The purpose of this study was to describe the residential movement patterns of a birth cohort of children in Ontario, Canada with a variety of complex and/or chronic conditions diagnosed in the first two years of life and to compare them to otherwise healthy children. The study used population-level administrative data to determine the aggregate patterns of movement at the level of the neighbourhood in families of young children and explored whether they are associated with different disease cohorts. Specifically, this study aimed to answer the following research questions:

(1) Are families of children with a complex and/or other chronic condition in the first two years of life more likely to move residence within the province within 5 years of birth compared with families of healthy children?

(2) In families who move residence, are those with children with a complex and/or other chronic condition more likely to move to lower income neighborhoods compared with families of healthy children? and,

(3) Are families of children with a complex and/or other chronic condition who live > 80 km from a tertiary care hospital more likely to move residence closer to that specialized setting of care compared with families of healthy children?

We hypothesized that families of infants with a complex and/or other chronic condition are more likely to move, and that due to the financial strains on families and the resource needs of these children, those who move are more likely to move to poorer neighborhoods and closer proximity to tertiary care.

## Methods

### Overall design and setting and population

We conducted a retrospective birth cohort study (all in-hospital live births from April 1, 2002 – March 31, 2007, N = 667,502) in Ontario, Canada’s most populous province (13.5 million), a jurisdiction with universal health care insurance for all primary and acute care services and variable levels of public, private insurance and out-of-pocket payment for other health services such as medications, home health care and durable medical devices. We followed all children until age five (last follow-up, Mar 31, 2012). This study utilized linked health care administrative databases housed at the Institute for Clinical Evaluative Sciences (ICES) to monitor residential movement trends of groups of children in the first five years of life. Ethics approval for this study was received from the Institutional Review Boards of the Hospital for Sick Children, Sunnybrook Health Sciences Center and Laurentian University.

### Measures and data sources

Study cohorts were constructed utilizing diagnostic codes from hospital [Discharge Abstract Database (DAD)], emergency and same day surgery [The National Ambulatory Care Reporting System (NACRS)] and physician billing (the Ontario Health Insurance Plan) datasets. The data quality of the DAD and NACRS is monitored regularly by the Canadian Institute for Health Information. The Registered Persons Database (RPDB) contains demographic and vital statistic data for all Ontario residents eligible for public health insurance. Variables include a unique identifier, sex, date of birth and, where applicable, date of death. ICES uses a unique scrambled identifier which permits linkage of an individual’s records across all databases and time. Postal codes were linked to the 2006 Canadian Census to obtain mean neighborhood income quintile for each dissemination area (population 400–700 inhabitants) that are adjusted for both household-size and community size. Statistics Canada has constructed the quintiles within each Census Metropolitan Area (CMA) or Census Agglomeration (CA) using the following algorithm. Persons were classified as having low income if their total economic family income in the preceding census was below that year’s Statistics Canada low-income cut-off, which varied according to family size and CMA/CA size. Each Census Tract (CT)/Dissemination Area (DA) within the CMA/CA was then ranked according to the percentage of the population below the low-income cut-off, and the CTs/DAs were assigned to five groups such that each of the five groups of CTs/DAs contained approximately one-fifth of the total non-institutional population of the CMA/CA.

This ecological proxy methodology has been found to reliably estimate household income quintile [17] and is widely used in Canadian child health services research [18-20]. Race and ethnicity data are not routinely collected in Canadian datasets.

### Study cohorts

An accrual window of the first two years of life was utilized to construct study cohorts. Five different cohorts were constructed hierarchically so that each was mutually exclusive from the other:

(a) & (b) *Complex Chronic Conditions* were defined utilizing the framework developed by Feudtner et al. as “any medical condition that can be reasonably expected to last at least 12 months (unless death intervenes) and to involve either several different organ systems or 1 organ system severely enough to require specialty pediatric care and probably some period of hospitalization in a tertiary care centre” [21,22]. This framework has been operationalized into a series of International Classification of Diseases (ICD) diagnoses (subdivided into nine organ system categories) for identifying CCCs using hospital discharge abstracts. For the purpose of this study, CCCs were subdivided into those affecting a single (single CCCs) versus multiple body systems (multiple CCCs), as those affecting multiple body systems are associated with increased health care use [23]. All hospital records from the first two years of life were used to define CCCs.

(c) *Low birthweight (LBW) infants*: This group included all infants with a birthweight < 2500 g without a CCC during the two year accrual window. This cohort was chosen as some of them (although not all) may develop neurodevelopmental disabilities and consequently complex health issues without necessarily a diagnosis of a CCC.

(d) *Asthma/Recurrent wheeze (A/RW)*: Children diagnosed with asthma before age 2 years without a CCC during those years were enrolled. This cohort was chosen to represent a common condition that is chronic but not usually considered complex, as it is usually managed in the community setting. We utilized the validated case definition of asthma which defines asthma as ≥ 2 outpatient visits and/or ≥ 1 hospitalization for asthma within two years. The Ontario Asthma Surveillance Information System (OASIS) has shown this definition to have up to 98% sensitivity and 91% specificity for diagnosing asthma [24]. Although it can be difficult to diagnose asthma accurately in very young children, recent chart validation suggests that administrative data can be as accurate in identifying asthma in this age group as in older children [25].

(e) *Healthy Children*: These included all other eligible children with a birth weight of ≥ 2500 grams who did not develop a CCC or asthma/recurrent wheeze or a non-newborn hospitalization during the first two years of life. This group was used for comparison in all analysis.

We excluded those children who were not Ontario residents at birth (N = 7739), those who died or moved out of Ontario prior to their second birthday (N = 6488), those without recorded birth weights (N = 294), with a birthdate outside the study accrual period (N = 1240) and otherwise healthy children with hospitalization before two years of age (N = 53,025, including 16 children not living in Ontario at index date of first hospitalization discharge) to arrive at a final cohort group of 598,716 children. Those who died or moved out of province from age 2–5 years were included and their last postal code used for all analyses.

### Outcomes

Movement of residence was defined as any change in postal code from birth to age 2–5 years. For children who had moved multiple times, the most recent postal code was used in the primary analyses. A drop in SES was defined as a decrease in income quintile from birth to age 2–5 years, or, for those in the lowest SES quintile, no change. Movement close to care was defined as movement from > 80 km from one of Ontario’s four academic pediatric hospitals [(The Hospital for Sick Children (Toronto, ON), Children’s Hospital of Eastern Ontario (Ottawa, ON), Children’s Hospital of Western Ontario (London, ON), McMaster Children’s Hospital (Hamilton, ON)], to ≤ 80 km from these institutions. A driving distance of 80 km was designated based on previous literature to reflect reasonable limitations of commuting distances [12], and was ascertained using mapping software that utilized road network analysis to calculate actual driving distance from the centre of the child’s enumeration or dissemination area (for most urban or suburban areas this would approximate one block). Since an 80 km categorical cut-off is somewhat arbitrary, we also measured this outcome as a continuous variable.

### Analysis

For univariate analyses, comparisons were made across groups using the chi-square test on percentages (categorical data) or the Kruskal-Wallis test on medians (continuous data). Multivariable analysis using logistic regression adjusting for potential confounders (gender, birth income quintile, and rurality) was use to measure the effect of chronic disease on residential movement or change in SES (for those that moved) by study cohort. Rurality was measured with the Rurality Index of Ontario (RIO) [26], a continuous measure of rurality formulated for health policy planning and research [27,28] ranging from 0 (least rural) to 100 (most rural). The RIO consists of both generic components of rurality such as population dispersion, remoteness and social factors as well as health-specific components such as distance to referral centers and ratios of population to family physicians. Separate sensitivity analyses were conducted including otherwise healthy children in Ontario who had a hospitalization in the first two years of life, and also excluding children who moved out of Ontario prior to age five years.

## Results

Of the 598,716 children included in the cohort study, 15,207 had a single CCC, 3,600 had multiple CCCs, 33,206 were LBW without a CCC, 57,137 had A/RW and 489,566 were healthy controls. Death during the outcome period (age 2–5 years) occurred in 230 children, of whom 97 had a CCC. Disparities in income quintile among study cohorts were apparent both at birth and at the end of the 5 year study period (Table 1). At birth, lower SES groups predominated in all disease cohorts. The proportion of children in the lowest income quintile ranged from 23.2% (healthy controls) to 25.8% (for the LBW group), but all chronic disease cohorts had a higher percentage of children in the lower birth income quintile than healthy controls (p < .001). Children across all cohorts showed increases in income quintile by age five. However, this effect was slightly more pronounced in the healthy controls, resulting in a widening of income disparities. By age five, the most common income category for single CCC, multiple CCC and LBW groupings was still the lowest quintile (quintile 1), compared with quintile 4 for asthma/recurrent wheeze and healthy controls.

**Table 1:** Characteristics of entire cohort at baseline (discharge from newborn hospitalization) and end of study period

	**Single CCC N = 15,207**		**Multiple CCCs N = 3,600**		**LBW N = 33,206**		**A/RW N = 57,137**		**Healthy controls N = 489,566**		**P-value**
	**Birth**	**End of period**	**Birth**	**End of period**	**Birth**	**End of period**	**Birth**	**End of period**	**Birth**	**End of period**	
**Gender, # (%)**											
Female	6527 (42.9%)		1602 (44.5%)		17,584 (52.9%)		20,920 (36.6%)		246,923 (50.4%)		<.001
**Neighbourhood Income Quintile, # (%)**											
Quintile 1 (lowest)	3751 (24.7%)	3211 (21.1%)	929 (25.8%)	800 (22.2%)	8564 (25.8%)	7446 (22.4%)	13,405 (23.5%)	11,752 (20.6%)	113,409 (23.2%)	93,276 (19.1%)	<.001**
Quintile 2	3184 (20.9%)	2932 (19.3%)	749 (20.8%)	650 (18.1%)	7111 (21.4%)	6553 (19.7%)	11,555 (20.2%)	10,714 (18.8%)	98,180 (20.1%)	90,080 (18.4%)	<.001**
Quintile 3	2982 (19.6%)	3014 (19.8%)	755 (21.0%)	769 (21.4%)	6585 (19.8%)	6631 (20.0%)	11,837 (20.7%)	11,870 (20.8%)	98,004 (20.0%)	99,001 (20.2%)	<.001**
Quintile 4	2869 (18.9%)	3170 (20.9%)	618 (17.2%)	716 (19.9%)	6092 (18.4%)	6736 (20.3%)	11,246 (19.7%)	12,309 (21.5%)	97,584 (19.9%)	109,270 (22.3%)	<.001**
Quintile 5 (highest)	2421 (15.9%)	2880 (18.9%)	549 (15.2%)	665 (18.5%)	4854 (14.6%)	5840 (17.6%)	9094 (15.9%)	10,492 (18.4%)	82,389 (16.8%)	97,939 (20.0%)	<.001**
**# of moves, median (IQR)**	--	1 (0,1)	--	1 (0,1)	--	1 (0,1)	--	0 (0,1)	--	1 (0,1)	<.001
Rurality (RIO)*, median (IQR)	6 (4–15)	6 (4–19)	6 (4–13)	6 (4–19)	5 (4–11)	6 (4–12)	6 (4–11)	6 (4–12)	6 (4–14)	6 (4–19)	<.001**
**#, % > 80 km from specialized hospital**	3,225 (21.2%)	3,459 (22.8%)	723 (20.1%)	759 (21.1%)	6,126 (18.4%)	6,677 (20.1%)	10,093 (17.7%)	10,691 (18.7%)	98,511 (20.1%)	106,199 (21.7%)	<.001**
**Distance (in km) between residence and specialized hospital, median (IQR)**	30 (13, 64)	32 (15, 68)	29 (12, 61)	31 (15, 64)	26 (13, 55)	29 (15, 60)	28 (15, 54)	30 (16, 56)	28 (13, 60)	30 (15, 65)	<.001

*Rurality Index of Ontario (RIO) [26], a continuous measure of rurality formulated for health policy planning and research [27,28] ranging from 0 (least rural) to 100 (most rural) using Census 2006. The RIO consists of both distinct generic components of rurality such as population dispersion, remoteness and social factors as well as components specific to health such as distance to referral centers and ratios of population to family. physicians. *CCC* complex chronic condition(s), *LBW* low birth weight, *A/RW* asthma/recurrent wheeze. ** P < .001 both for comparisons at birth and comparisons at end of study period. All P-values indicate tests of differences across groups.

**Figure 1:**
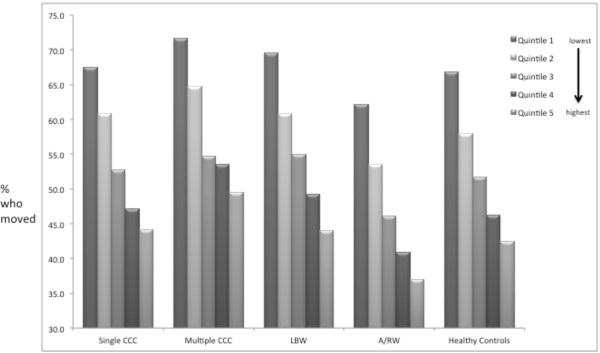
**Rates of movement across chronic condition groupings and income quintile.** Quintiles are arranged in ascending order. CCC = complex chronic condition(s), LBW = low birth weight, A/RW = asthma/recurrent wheeze.

**Figure 2:**
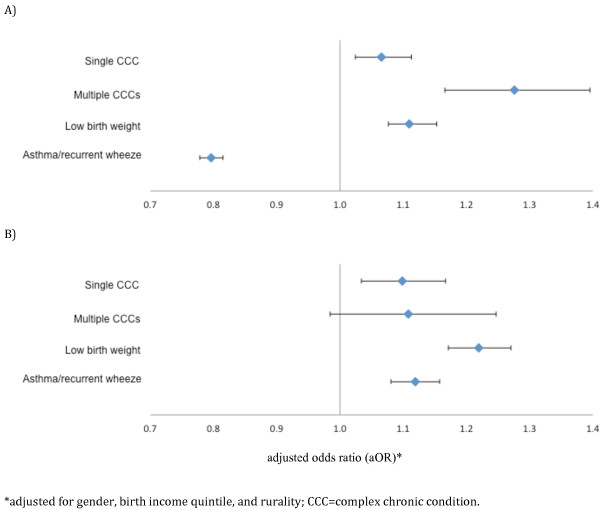
Adjusted* odds ratios (and 99% confidence intervals) of movement (PANEL A) or among those whom moved, a drop in income quintile (or for those in the lowest income quintile, no change in income quintile) (PANEL B) compared with healthy controls.

Overall rates of residential movement by age 5 were slightly higher in complex chronic disease groups (60.2% for multiple CCCs and 55.6% for single CCCs) and LBW (57.3%) but not for A/W (49.0%) when compared with 53.8% for healthy controls (p < .001) (Table 2). However these differences across study cohorts were much smaller in magnitude than differences in movement across birth SES (Figure 1). Children in the lowest income quintile were more likely to move across all cohorts (ranging from 62.2% for A/RW to 71.7% for multiple CCCs). Less than half of children in the highest quintile moved in all the cohorts. All cohorts showed upward mobility across income quintiles over the course of the observation period (Table 3). The median distance that the cohorts moved was not large in any group ranging from 3.5 kilometers (multiple groups) to 5.0 kilometers for LBW in the highest income quintile (Table 4). In multivariable modeling adjusting for gender, birth income quintile, and rurality, all the chronic disease cohorts except A/RW were more likely to move compared with healthy controls, and all chronic disease cohorts were slightly more likely to move to a lower income quintile neighborhood compared with healthy controls (adjusted odds ratio [aOR] ranging from 1.1 (99% confidence interval [CI]: 1.03 to 1.17) for single CCC to 1.2 (99% CI: 1.17 to 1.26) for LBW) (Figure 2). Odds ratio estimates were similar with inclusion of otherwise healthy children with hospitalizations in the first two years of life into the model, and excluding those who moved out of Ontario prior to age five years [n = 8,768 (1.46%), of whom 1,260 (14.3%) returned by the end of the study period]. For those living >80 km from specialized care, there was no significant difference in movement closer to specialized care in families with CCCs.

**Table 2:** Characteristics of children who moved by disease cohort

	**Single CCC**	**Multiple CCCs**	**LBW**	**A/RW**	**Healthy controls**	P-value*
	**N = 15,207**	**N = 3,600**	**N = 33,206**	**N = 57,137**	**N = 489,566**	
n,(%**)	8,467 (55.6%)	2,167 (60.2%)	19,036 (57.3%)	27,995 (48.9%)	263,576 (53.8%)	<.001
Female, n (%**)	3,573 (54.7%)	953 (59.5%)	10,077 (57.3%)	10,104 (48.3%)	132,504 (53.7%)	<.001
Male, n (%**)	4,894 (56.4%)	1,214 (60.8%)	8,959 (57.3%)	17,851 (49.3%)	131,072 (54.0%)	<.001
Neighbourhood Income Quintile at birth, # (%*)						
Quintile 1 (lowest)	2,531 (67.5%)	666 (71.7%)	5,958 (69.6%)	8,341 (62.2%)	75,794 (66.8%)	<.001
Quintile 2	1,940 (60.9%)	485 (64.8%)	4,321 (60.8%)	6,196 (53.6%)	56,927 (58.0%)	<.001
Quintile 3	1,575 (52.8%)	413 (54.7%)	3,619 (55.0%)	5,460 (46.1%)	50,661 (51.7%)	<.001
Quintile 4	1,350 (47.1%)	331 (53.6%)	3,002 (49.3%)	4,597 (40.9%)	45,219 (46.3%)	<.001
Quintile 5 (highest)	1,071 (44.2%)	272 (49.5%)	2,136 (44.0%)	3,361 (37.0%)	34,975 (42.5%)	<.001
> 80 km from specialized hospital at birth, n (%***)	1,728 (53.6%)	444 (61.4%)	3,428 (56.0%)	5,101 (50.5%)	50,690 (51.5%)	<.001

*P-values indicate tests of differences across groups.

**% of total group who moved at least once from Year 2–5.

***% based on children living > 80 km at baseline only.

*CCC* complex chronic condition(s), *LBW* low birth weight, *A/RW* asthma/recurrent wheeze.

**Table 3:** Changes in income quintile by disease cohort among children who have moved at 2–5 years of age

	**Single CCC**	**Multiple CCCs**	**LBW**	**A/RW**	**Healthy controls**	P-value*
	**N = 8,467**	**N = 2,167**	**N = 19,036**	**N = 27,955**	**N = 263,576**	
**Income Quintile Change**						
SES increases from birth, n (%)	3,461 (40.9%)	896 (41.4%)	7,774 (40.9%)	11,309 (40.4%)	113,031 (42.9%)	.0075
SES decreases from birth, n (%)	2,209 (26.1%)	560 (25.8%)	5,114 (26.9%)	7,162 (25.6%)	67,671 (25.7%)	<.001
SES no change from birth, n (%)	2,797 (33.0%)	711 (32.8%)	6,148 (32.3%)	9,484 (33.9%)	82,874 (31.4%)	<.001

*P values indicate tests of difference across groups.

*CCC* complex chronic condition(s), *LBW* low birth weight, *A/RW* asthma/recurrent wheeze.

**Table 4:** Distance of movement in kilometers by birth income quintile and cohort

	**Single CCC, median (IQR)**	**Multiple CCC, median (IQR)**	**LBW, median (IQR)**	**A/RW, median (IQR)**	**Healthy controls, median (IQR)**	P-value*
	**N = 8,467**	**N = 2,167**	**N = 19,036**	**N = 27,955**	**N = 263,576**	
**Birth Income Quintile**						
1 (lowest)	3.5 (0.9, 13.0)	3.5 (0.8, 11.6)	3.9 (0.9, 12.6)	3.5 (0.9, 10.8)	4.1 (1.1, 13.4)	<.001
2	3.6 (1.0, 12.2)	3.9 (0.9, 12.8)	4.1 (1.1, 12.2)	3.7 (1.0, 11.2)	4.4 (1.2, 13.6)	<.001
3	4.1 (1.1, 13.6)	4.6 (1.2, 14.0)	4.8 (1.3, 13.9)	3.7 (1.1, 11.5)	4.4 (1.2, 13.8)	<.001
4	4.4 (1.1, 13.9)	4.9 (1.2, 15.2)	4.5 (1.3, 13.2)	3.7 (1.0, 11.8)	4.5 (1.2, 14.4)	<.001
5 (highest)	4.3 (0.9, 15.3)	4.0 (1.4, 14.7)	5.0 (1.2, 15.7)	3.5 (0.8, 12.2)	4.4 (1.1, 14.7)	<.001

*P values indicate test of difference across groups.

*CCC* complex chronic condition(s), *LBW* low birth weight, *A/RW* asthma/recurrent wheeze.

## Discussion

This study is, to our knowledge, the first to examine longitudinally the socio-economic status of young children born with chronic conditions and their residential movement patterns. We found that these children were, on average, slightly poorer at birth and slightly more likely to move to a neighborhood with a lower income quintile or remain in the lowest income quintile neighborhood compared with healthy children. However, relative to the effects of chronic disease, socio-economic status seems to be a much stronger driver of movement. Children born into poverty are more likely to move than those born into relative wealth.

Our finding of slightly widening income disparities for families of children with chronic disease compared with healthy children over the course of the lifespan were from a jurisdiction with universal health insurance, indicating that insurance status alone may not explain the financial strain on families of children with chronic disease. Potential explanations for neighbourhood income disparities at birth include poorer maternal nutrition, barriers to access to antenatal care and higher risk of certain congenital anomalies [29,30]. Potential explanations for widening disparities after birth include direct financial impacts on families from incomplete public insurance coverage (e.g. not all medications are publicly funded in Ontario), and indirect costs of unpaid caregiving on families. One recent analysis of the National Survey of Children with Special Health Care Needs (CSHCN) in the United States estimated that 54% of families of more complex CSHCN reported that a member stopped working because of the child’s health [31]. Findings from the Participation and Activity Limitation Survey (2006) showed that a relationship existed between whether a Canadian family was deemed to be living in poverty and whether a child with a disability was present in the household. Furthermore, the severity of the child’s disability had a tremendous impact with families with children with a severe to very severe disability reporting financial difficulties more than three times greater than families of children with mild to moderate disabilities [32].

The findings contribute to a growing understanding of health selective immobility and health selective movement. Previous evidence shows that the healthiest move and the unhealthy stay. Gatrell [33] summarizes this idea of the selectivity of movement as “[T]hose with poorer health may be less likely to move because of the physical upheaval and mental stress involved with the move”. In contrast, we found that chronic and/or complex disease populations were slightly more likely to move residence. It is important to note that the selective migration hypothesis is usually studied in international migration and not regional movement. Another possible explanation is that children with CCC and their families are a very heterogenous group in terms of illness and family characteristics, however, we were unable to explore this due to the availability of limited variables in the administrative data set that was used. It may be that children with less severe CCC and their families fared better over the study period and experienced upward SES mobility which provides an example of health selective movement. Whereas children with very severe limitations and their families fared less well, and therefore stayed in the lowest income bracket (health selective immobility) or experienced downward SES mobility.

Our findings have some similarities and some differences with existing studies examining the links between illness/disability and movement, which have been primarily related to persons with HIV/AIDS. Berk et al. found that a nationally representative sample of persons with HIV/AIDS in the United States were not only more likely to move than those without HIV/AIDS, but also more likely to move further (e.g. to a neighbouring state) [34]. For children, we found modest differences in the rates of movement for those children with complex chronic diseases compared with a healthy control group, but not for children with a less complex condition (A/RW), and the amount of distance moved was similar across cohorts. Participants in a qualitative study of the migratory experiences of people with HIV/AIDS discussed a number of reasons behind their decision to move including accessing health care services, accessing social support networks, moving nearer to family, and socio-economic concerns [11]. In our research we did not find that accessing specialized (tertiary) care was a significant motivator for the residential movement of families with CCC. Several reasons may explain this somewhat surprising finding. Firstly, our study utilized population-level data which limited our ability to explore the reasons why families moved. Secondly, for these studies concerning chronic illness and movement, the focus is on the individual living with HIV/AIDS. In our study, focusing on movement of the family unit rather than the individual and experiencing a more diverse set of chronic conditions, there is inevitably more complexity involved with decision-making about movement.

Implications of our research may include continuity of health care. Much effort has been made in recent years to promote the concept of a well-developed primary care Medical Home as ideal for the care of (CSHCN) [35]. One of the central elements of this concept is continuity of care defined as “the relationship between a single practitioner and a patient that extends beyond specific episodes of illness or disease” [36]. Previous cross-sectional data has demonstrated that disparities in access to a medical home are determined by geography, race/ethnicity, income, health insurance status, and severity of the child's condition [37,38]. In our study, most families moved short distances, so we do not know if this had any disruption on continuity of care, particularly in areas with well-established transportation networks. However, at least for some families, disparities in access to a medical home may be exacerbated over the course of the lifespan of children with economic (low income) and medical (complex needs) vulnerabilities as movement may lead to increased risk of discontinuity of care, and consequently, the potential loss of crucial information and weakening of therapeutic relationships between the practitioner and patient and their family. This could result in the impaired ability of a practitioner to provide the best possible care, and makes it more difficult for the child and family to manage the complex and/or chronic condition. Follow-up research is needed to determine the consequences of residential movement for the health and well being of children with CCC.

Several limitations to this study should be noted. First, the definition of our chronic disease cohorts utilized algorithms previously used in health services research that include varying severity and complexity and that have imperfect sensitivity and specificity (e.g. asthma). Some of the children meeting these definitions may have been misclassified as having a chronic disease (e.g. a child with recurrent wheeze who does not develop asthma or a child who is LBW with a normal neurodevelopmental outcome) and we may have labeled children with important chronic conditions that do not lead to hospitalization such as autism as healthy. Both of these potential sources of misclassification would likely provide bias to the null hypothesis. Second, we defined neighborhood at the level of the dissemination area, the smallest geographic unit for which Canadian census data is available. Although previous studies have demonstrated good correlation between these data and individual household income, the precision of this ecologic methodology for geocoding drops in rural areas, leading to misclassification of income status, and a bias to the null. Third, although our sample size was robust for most analyses, we had small numbers for some specific subgroups, such as very low birth weight migrators who were born > 80 km from hospital; in subgroups with large sample sizes, statistically significant findings of small differences may not be meaningful. Fourth, we were limited in our analyses to covariates that were accessible in Ontario’s linked health administrative databases and did not explore other potentially important predictors of family movement such as ethnicity, family characteristics (e.g. immigration status or family education level), and out-of-pocket expenses. Lastly, we have no knowledge of the reasons for residential movement among families in this study or how it may have affected family functioning. Previous literature has documented that reasons for movement are usually multi-factorial, a combination of social, economic, religious, political and personal ‘push’ and ‘pull’ factors [39]. Further research using qualitative methodologies is necessary to explore the reasons why families with children with complex chronic conditions are more likely to move neighbourhoods and how important this residential movement is in terms of healthcare access.

## Abbreviations

CSHCN: Children with special health care needs; CCC: Complex chronic condition(s); LBW: Low birth weight; A/RW: Asthma/recurrent wheeze; SES: Socio-economic status; CCC: Complex chronic conditions; LBW: Low birthweight; A/RW: Asthma/recurrent wheeze; CSHCN: Children with special health care needs.

## Competing interest

Dr. Cohen and Guttmann have received funding for this project from the Norman Saunders Complex Care Initiative administered through the SickKids Foundation. The Institute for Clinical Evaluative Sciences (ICES) is a nonprofit organization funded by the Ontario Ministry of Health and Long-Term Care (MOHLTC) with provision of population-based data. No endorsement by ICES or the Ontario MOHLTC is intended or should be inferred.

## Authors’ contributions

EC, NY, KL AND AG were involved in study conception and design. EC, NY, JG, KL and AG were involved in analysis and interpretation of data. EC and AG drafted the article. All authors were involved in revisions of the manuscript for important intellectual content and all authors read and approved of the final version.

## References

[B1] Wise PH The rebirth of pediatrics Pediatrics 2009 123 1 413 416 10.1542/peds.2008-3254 19117909

[B2] U.S. Department of Health and Human Services Healthy people 2020: improving the health of Americanshttp://healthypeople.gov/2020/default.aspx

[B3] Patterson JM Leonard BJ Titus JC Home care for medically fragile children: impact on family health and well-being J Dev Behav Pediatr 1992 13 4 248 255 1506462

[B4] Baker BL McIntyre LL Blacher J Crnic K Edelbrock C Low C Pre-school children with and without developmental delay: behaviour problems and parenting stress over time J Intellect Disabil Res 2003 47 Pt 4–5 217 230 12787154 10.1046/j.1365-2788.2003.00484.x

[B5] Ratliffe CE Harrigan RC Haley J Tse A Olson T Stress in families with medically fragile children Issues Compr Pediatr Nur 2002 25 3 167 188 10.1080/01460860290042558 12230829

[B6] Teague BR Fleming JW Castle A Kiernan BS Lobo ML Riggs S Wolfe JG “High-tech” home care for children with chronic health conditions: a pilot study J Pediatr Nurs 1993 8 4 226 232 8410643

[B7] Newacheck PW Halfon N Prevalence and impact of disabling chronic conditions in childhood Am J Public Health 1998 88 4 610 617 10.2105/AJPH.88.4.610 9551003 PMC1508436

[B8] Dummer TJ Health geography: supporting public health policy and planning CMAJ 2008 178 9 1177 1180 10.1503/cmaj.071783 18427094 PMC2292766

[B9] Cummings KM Becker MH Maile MC Bringing the models together: an empirical approach to combining variables used to explain health actions J Behav Med 1980 3 2 123 145 10.1007/BF00844986 7420418

[B10] Melnyk KA Barriers: a critical review of recent literature Nurs Res 1988 37 4 196 201 3293024

[B11] Elmore K The migratory experiences of people with HIV/AIDS (PWHA) in Wilmington, North Carolina Health Place 2006 12 4 570 579 10.1016/j.healthplace.2005.08.009 16168702

[B12] Yantzi N Rosenberg MW Burke SO Harrison MB The impacts of distance to hospital on families with a child with a chronic condition Soc Sci Med 2001 52 12 1777 1791 10.1016/S0277-9536(00)00297-5 11352405

[B13] Weeks JR Population: an introduction to concepts and issues 2012 11 California: Wadsworth, Cengage Learning

[B14] Meyer JW Cromley EK Caregiving environments and elderly residential mobility Prof Geogr 1989 41 4 440 450 10.1111/j.0033-0124.1989.00440.x

[B15] Wen M Fan J Jin L Wang G Neighbourhood effects on health among migrants and natives in Shanghai, China Health Place 2010 16 452 460 10.1016/j.healthplace.2009.12.001 20060767

[B16] Lu Y Test of the ‘healthy migrant hypothesis’: a lonngitudinal analysis of health selectivity of internal migration in Indonesia Soc Sci Med 2008 67 1331 1339 10.1016/j.socscimed.2008.06.017 18639967

[B17] Mustard CA Derksen S Berthelot JM Wolfson M Assessing ecologic proxies for household income: a comparison of household and neighbourhood level income measures in the study of population health status Health Place 1999 5 2 157 171 10.1016/S1353-8292(99)00008-8 10670997

[B18] Kozyrskyj AL Dahl ME Chateau DG Mazowita GB Klassen TP Law BJ Evidence-based prescribing of antibiotics for children: role of socioeconomic status and physician characteristics CMAJ 2004 171 2 139 145 10.1503/cmaj.1031629 15262882 PMC450362

[B19] Kozyrskyj AL Mustard CA Simons FE Socioeconomic status, drug insurance benefits, and new prescriptions for inhaled corticosteroids in schoolchildren with asthma Arch Pediatr Adolesc Med 2001 155 11 1219 1224 10.1001/archpedi.155.11.1219 11695930

[B20] Wang C Guttmann A To T Dick PT Neighborhood income and health outcomes in infants: how do those with complex chronic conditions fare? Arch Pediatr Adolesc Med 2009 163 7 608 615 10.1001/archpediatrics.2009.36 19581543

[B21] Feudtner C Chirstakis D Connell F Pediatric deaths attributable to complex chronic conditions: a population-based study of Washington State, 1980–1997 Pediatrics 2000 106 205 209 10888693

[B22] Feudtner C Silveira M Christakis D Where do children with complex chronic conditions die? Patterns in Washington State, 1980–1998 Pediatrics 2002 109 4 656 660 10.1542/peds.109.4.656 11927711

[B23] Cohen E Berry JG Camacho X Anderson G Wodchis W Guttmann A Patterns and costs of health care use of children with medical complexity Pediatrics 2012 130 6 e1463 e1470 10.1542/peds.2012-0175 23184117 PMC4528341

[B24] To T Dell S Dick P Cicutto L Harris J Tassoudji M Duong-Hua M Burden of Childhood Asthma 2004 Toronto, Ontario: ICES

[B25] To T Dell S Dick P Cicutto L Harris J MacLusky I Tassoudji M Case verification of children with asthma in Ontario Pediatr Allergy Immunol 2006 17 69 76 10.1111/j.1399-3038.2005.00346.x 16426258

[B26] Kralji B **Measuring “rurality” for purposes of health-care planning** Ont Med Rev 2000 10 33 52

[B27] Guilcher SJ Munce SE Couris CM Fung K Craven BC Verrier M Jaglal SB **Health care utilization in non-traumatic and traumatic spinal cord injury: a population-based study** Spinal Cordv 48 1 45 50 19546877 10.1038/sc.2009.78

[B28] Jaglal SB Munce SE Guilcher SJ Couris CM Fung K Craven BC Verrier M **Health system factors associated with rehospitalizations after traumatic spinal cord injury: a population-based study** Spinal Cord 2009 47 8 604 609 10.1038/sc.2009.9 19274059

[B29] Agha MM Glazier RH Moineddin R Moore AM Guttmann A **Food fortification and decline in the prevalence of neural tube defects: does public intervention reduce the socioeconomic gap in prevalence?** Int J Environ Res Public Health 2013 10 4 1312 1323 10.3390/ijerph10041312 23538728 PMC3709319

[B30] Agha MM Glazier RH Moineddin R Moore AM Guttmann A **Socioeconomic status and prevalence of congenital heart defects: does universal access to health care system eliminate the gap?** Birth Defects Res A Clin Mol Teratol 2011 91 12 1011 1018 10.1002/bdra.22857 22002854

[B31] Kuo D Cohen E Agrawal R Berry JG Casey PH **National profile of caregiver challenges of more-complex children with special health care needs** Arch Pediatr Adolesc Med 2011 in press 10.1001/archpediatrics.2011.172 PMC3923457 22065182

[B32] Canada S Participation and activity limitation survey 2006: families of children with disabilities in Canada 2008 Ottawa: Statistics Canada

[B33] Gatrell AC Mobilities and health 2011 Lancaster, UK: Ashgate

[B34] Berk ML Schur CL Dunbar JL Bozzette S Shapiro M **Short report: migration among persons living with HIV** Soc Sci Med 2003 57 6 1091 1097 10.1016/S0277-9536(02)00487-2 12878108

[B35] Homer CJ Klatka K Romm D Kuhlthau K Bloom S Newacheck P Van Cleave J Perrin JM **A review of the evidence for the medical home for children with special health care needs** Pediatrics 2008 122 4 e922 e937 10.1542/peds.2007-3762 18829788

[B36] Haggerty JL Reid RJ Freeman GK Starfield BH Adair CE McKendry R **Continuity of care: a multidisciplinary review** BMJ 2003 327 7425 1219 1221 10.1136/bmj.327.7425.1219 14630762 PMC274066

[B37] Singh GK Strickland BB Ghandour RM van Dyck PC **Geographic disparities in access to the medical home among US CSHCN** Pediatrics 2009 124 Suppl 4 S352 S360 19948599 10.1542/peds.2009-1255E

[B38] Strickland BB Singh GK Kogan MD Mann MY van Dyck PC Newacheck PW **Access to the medical home: new findings from the 2005–2006 national survey of children with special health care needs** Pediatrics 2009 123 6 e996 e1004 10.1542/peds.2008-2504 19482751

[B39] Norton W Human geography 2009 7 Toronto, Ontario: Oxford University Press

